# High expression of Ki-67 is an independent favorable prognostic factor for esophageal small cell carcinoma

**DOI:** 10.18632/oncotarget.19426

**Published:** 2017-07-21

**Authors:** Han-Yu Deng, Zi-Hang Chen, Zhi-Qiang Wang, Yun-Cang Wang, En-Min Li, Li-Yan Xu, Yi-Dan Lin, Long-Qi Chen

**Affiliations:** ^1^ Department of Thoracic Surgery, West China Hospital of Sichuan University, Chengdu, Sichuan 610041, China; ^2^ Lung Cancer Center, West China Hospital of Sichuan University, Chengdu, Sichuan 610041, China; ^3^ Department of Pathology, West China Hospital, Sichuan University, Chengdu, Sichuan 610041, China; ^4^ The Key Laboratory of Molecular Biology for High Cancer Incidence Coastal Chaoshan Area and Department of Biochemistry and Molecular Biology, Shantou University Medical College, Shantou, Guangdong 515041, China; ^5^ The Key Laboratory of Molecular Biology for High Cancer Incidence Coastal Chaoshan Area and Institute of Oncologic Pathology, Shantou University Medical College, Shantou, Guangdong 515041, China

**Keywords:** esophagus, small cell carcinoma, Ki-67, prognosis

## Abstract

**Background:**

The prognostic value of Ki-67 expression in small cell carcinoma of the esophagus (SCCE) has not been explored in any previous studies. Therefore, we conducted this retrospective study to investigate the prognostic role of Ki-67 in SCCE for the first time.

**Results:**

A total of 44 patients were included for analysis. The baseline clinicopathological data of these SCCE patients shared similar characteristics with previous studies. Ten patients were at stage I, 17 at stage II, and the remaining 17 were at stage III. Postoperatively, 23 patients received adjuvant therapy. Twenty-eight patients were found to have a high expression of Ki-67 (> 50%). After a median follow-up time of 54.8 months, the median survival time of those patients was 22.1 months. Early TNM stage, application of adjuvant therapy, and high expression of Ki-67 (Hazard Ratio = 0.314, 95% CI: 0.127–0.774; *P* = 0.012) were found to be favorable prognostic factors of patients with SCCE. In subgroup analysis, adjuvant therapy could only bring significant survival benefit for patients with high expression of Ki-67 (*P* = 0.008).

**Materials and Methods:**

Patients undergoing esophagectomy with lymphadenectomy for SCCE from January 2009 to January 2015 in our department were retrospectively analyzed. Data for analysis included demographic data, pathologic findings, tumor stage, adjuvant therapy, and survival time as well as Ki-67 index.

**Conclusions:**

This study suggested that high expression of Ki-67 may not only serve as a favorable prognostic factor of SCCE but also an indication of providing adjuvant therapy for SCCE patients with surgical resection.

## INTRODUCTION

Esophageal cancer is the eighth most common malignant tumors and the sixth most common cause of death from cancer worldwide [1]. However, small cell carcinoma of the esophagus (SCCE), as one kind of neuroendocrine carcinoma originated from esophagus characterized as high grade neuroendocrine carcinoma with a mitotic count of > 20 per 10 high power fields and/ or a Ki-67 index > 20% [2], was reported to account for only 0.5% to 5.9% of all esophageal cancer in Chinese patients and 1% to 2.8% in Western patients [3]. Due to its rarity, little has been known about the clinicopathological and immunohistochemical characteristics of SCCE, and the prognostic factors of SCCE were far from established yet.

Ki-67, which is a 359-kD non-histone nuclear antigen identified by monoclonal antibody Ki-67 [4], is found to be present during all active phases of cell cycle but absent from resting cells. Therefore, it served as a proliferation marker [5]. In clinical pathology, the fraction of Ki-67-positive tumor cells, also called the Ki-67 labelling index, was routinely applied to estimate the growth fraction of a tumor [5–7]. Ki-67 labelling index has been intensively investigated as prognostic marker for various tumors. In most of the previous studies, high Ki-67 index was found to be significantly correlated with poor prognosis in patients with tumors, such as lymphoma [8], bladder cancer [9], lung cancer [10, 11], liver malignancies [12], and even neuroendocrine tumors [7]. However, there were also studies showing that high Ki-67 index was an independent favorable prognostic marker in colorectal cancer [6]. Apart from the role of prognostic marker, Ki-67 index was also applied to neuroendocrine neoplasms for classification and grading by 2010 World Health Organization classification (WHO) [2]. Herein, neuroendocrine carcinoma was graded as G3 with a Ki-67 index of more than 20%. Even though SCCE, as one kind of neuroendocrine carcinoma, was characterized as a Ki-67 index of more than 20%, the prognostic value of Ki-67 index for SCCE was not investigated in any previous study due to its rarity. Therefore, in our current study, we tried to explore the prognostic value of high Ki-67 expression (> 50%) compared with low Ki-67 expression (≤ 50%) in SCCE patients with surgical resection. To our knowledge, this is the first study focusing on the prognostic value of Ki-67 expression of SCCE.

## RESULTS

### Patient characteristics

From January 2009 to January 2015, a total of 3212 patients were recorded to undergo esophagectomy with lymphadenectomy for esophageal cancer in our department, 44 (accounting for 1.4% of all esophageal cancers) of which were SCCE. The baseline characteristics of those SCCE patients were shown in Table 1. The mean age of those patients were 59.9 ± 8.7 years old (ranging from 42 to 76 years) with a male to female ratio of 3:1. The majority of the esophageal NEC were located in the middle (61.4%) segment of the esophagus. For pathological findings, 15 patients were found to have mixed SCCE, which were all mixed with esophageal squamous cell carcinoma. Six patients were proved to have lymphovascular invasion. Because those patients were preoperatively diagnosed as clinical stage I or stage II disease, none of them received any neoadjuvant therapy, and all of them were intended for radical resection with two-field lymphadenectomy. Because of the historical background of choosing left thoracotomy as a predominant approach to esophageal cancer in China, 40 patients underwent left thoracotomy with two-field lymphadenectomy, while 4 patients received right thoracotomy with two-field lymphadenectomy. All those patients underwent R0 resection with negative resection margins. According to the 2009 AJCC TNM staging system, ten patients were at pathological stage I, 17 at stage II, and 17 at stage III. Postoperatively, more than half of those patients received adjuvant therapy (chemotherapy and/or radiotherapy). All those patients were estimated to have a Ki-67 index of more than 20%, and twenty-eight patients were found to have a Ki-67 index of more than 50% (high expression of Ki-67).

**Table 1:** The baseline characteristics of those patients with small cell carcinoma of the esophagus

Characteristics	No. in group (%)
Mean age (range)	59.9 ± 8.7 (42–76) years
Gender	
Male	33 (75.0)
Female	11 (25.0)
Tumor location	
Upper	4 (9.1)
Middle	27 (61.4)
Lower	13 (29.5)
Histology homology	
Pure	29 (65.9)
Mixed	15 (34.1)
Lymphovascular invasion	
Yes	6 (13.6)
No	38 (86.4)
Pathological TNM stage	
Stage I	10 (22.7)
Stage II	17 (38.6)
Stage III	17 (38.6)
pT stage	
T1	13 (29.5)
T2	16 (36.4)
T3	12 (27.3)
T4	3 (6.8)
pN stage	
N0	16 (36.4)
N1	16 (36.4)
N2	10 (22.7)
N3	2 (4.5)
Surgical approaches	
Left thoracotomy	40 (90.9)
Right thoracotomy	4 (9.1)
Adjuvant therapy	
Yes	23 (52.3)
No	21 (47.7)
Ki-67	
Low expression (≤ 50%)	16 (36.4)
High expression (> 50%)	28 (63.6)

### Clinicopathological characteristics of Ki-67 in SCCE

The correlation between Ki-67 index and clinicopathological characteristics of SCCE was shown in Table 2. There was no obvious correlation between Ki-67 expression and the age, gender, tumor location, histology homology, Lymphovascular invasion, pT stage, and pN stage of SCCE. Moreover, the numbers of patients receiving adjuvant therapy were comparable between high Ki-67 expression group and low Ki-67 expression group.

**Table 2:** Correlation between Ki-67 index and clinicopathological characteristics in patients with small cell carcinoma of the esophagus

Characteristics	Low Ki-67 index, *N* (%)	High Ki-67 index, *N* (%)	*P* value
Mean age (years)	62.3 ± 8.1	58.5 ± 8.9	0.169
Gender			1.00
Male	12 (27.3)	21 (47.7)	
Female	4 (9.1)	7 (15.9)	
Tumor location			0.733
Upper	1 (2.3)	3 (6.8)	
Middle	11 (25.0)	16 (36.4)	
Lower	4 (9.1)	9 (20.5)	
Histology homology			0.764
Pure	11 (25.0)	18 (40.9)	
Mixed	5 (11.4)	10 (22.7)	
Lymphovascular invasion			0.868
Yes	14 (31.8)	24 (54.5)	
No	2 (4.5)	4 (9.1)	
Pathological TNM stage			0.838
Stage I	3 (6.8)	7 (15.9)	
Stage II	7 (15.9)	10 (22.7)	
Stage III	6 (13.6)	11 (25.0)	
pT stage			0.491
T1	4 (9.1)	9 (20.5)	
T2	7 (15.9)	9 (20.5)	
T3	5 (11.4)	7 (15.9)	
T4	0 (0)	3 (6.8)	
pN stage			0.545
N0	6 (13.6)	10 (22.7)	
N1	5 (11.4)	11 (25.0)	
N2	5 (11.4)	5 (11.4)	
N3	0 (0)	2 (4.5)	
Adjuvant therapy			0.690
Yes	7 (15.9)	14 (31.8)	
No	9 (20.5)	14 (31.8)	

### Survival and prognostic analysis

**Figure 1:**
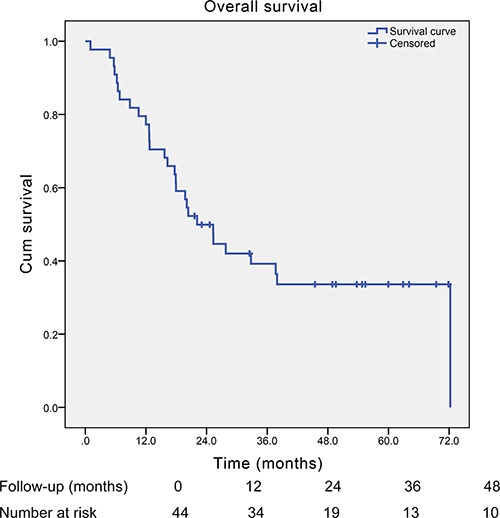
Kaplan-Meier curve of the overall survival of all those esophageal small cell carcinoma patients with surgical resection (median survival time: 22.1 months)

**Figure 2:**
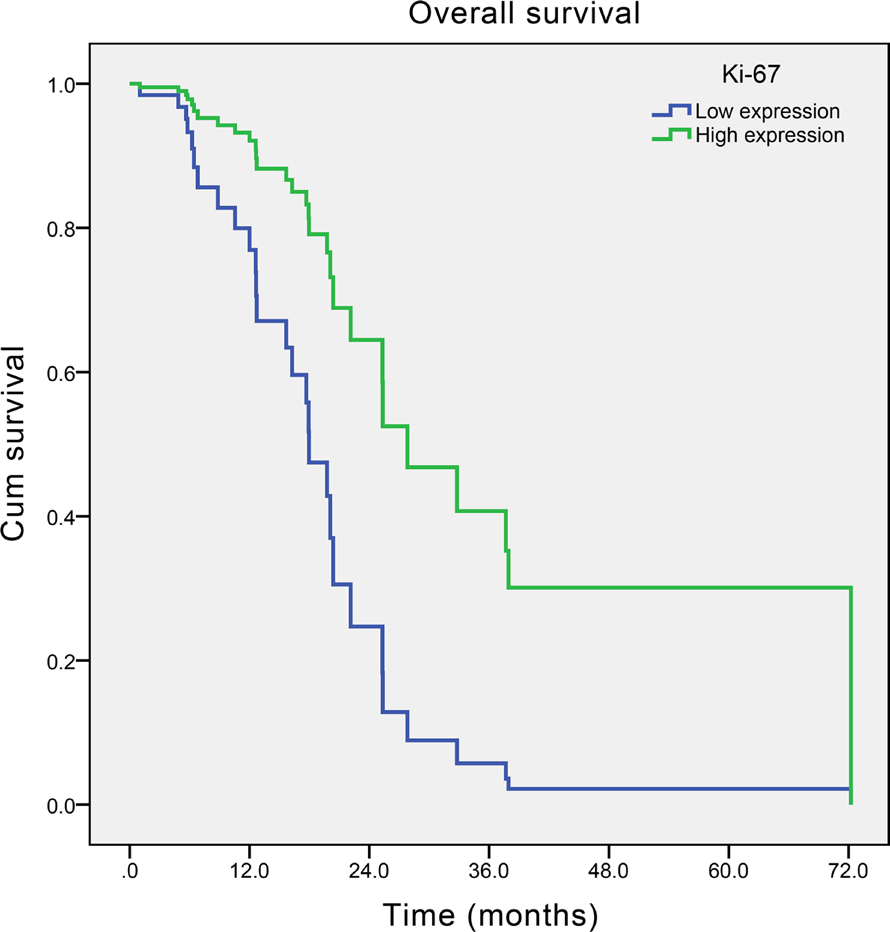
Adjusted survival curve by Cox's hazard regression model of overall survival of esophageal small cell carcinoma patients with different Ki-67 index (*P* = 0.012)

After a median follow-up time of 54.8 months, the median survival time of those SCCE patients was 22.1 months (95% Confidence Interval (CI): 15.3–28.9 months) (Figure 1). In the univariate analysis, only TNM stage and adjuvant therapy were found to significantly influence the overall survival of SCCE patients. However, in the multivariate analysis, apart from TNM stage and adjuvant therapy, Ki-67 index (Hazard Ratio (HR) = 0.314, 95% CI: 0.127–0.774; *P* = 0.012, Figure 2) was also found to be an independent prognostic factor of patients with SCCE (Table 3).

**Table 3:** Univariate and multivariate analyses of survival for small cell carcinoma of the esophagus

Variables	Reference level	Univariate analysis		Multivariate analysis	
		HR (95%CI)	*P* value	HR (95%CI)	*P* value
Age		0.994 (0.952–1.036)	0.764	0.966 (0.918–1.017)	0.188
Gender					
Male	Female	1.298 (0.526–3.203)	0.572	0.835 (0.296–2.353)	0.733
Tumor location					
Middle segment	Upper segment	0.864 (0.252–2.958)	0.816	2.327 (0.630–8.600)	0.205
Lower segment		0.920 (0.243–3.485)	0.903	1.618 (0.392–6.678)	0.506
Histology homology					
Mixed	Pure	0.602 (0.270–1.342)	0.215	1.219 (0.442–3.363)	0.702
Lymphovascular invasion					
Yes	No	1.111 (0.385–3.205)	0.846	0.750 (0.224–2.513)	0.641
Pathological TNM stage					
Stage I	Stage III	0.133 (0.038–0.472)	0.002*	0.030 (0.006–0.152)	< 0.001*
Stage II		0.378 (0.166–0.860)	0.020*	0.234 (0.087–0.630)	0.004*
Adjuvant therapy					
Yes	No	0.392 (0.184–0.837)	0.016*	0.144 (0.049–0.417)	< 0.001*
Ki-67					
High expression (> 50%)	Low expression (≤ 50%)	0.631 (0.294–1.356)	0.238	0.314 (0.127–0.774)	0.012*

Note: HR = Hazard Ratio; CI = Confidence Interval; *P < 0.05.

**Figure 3:**
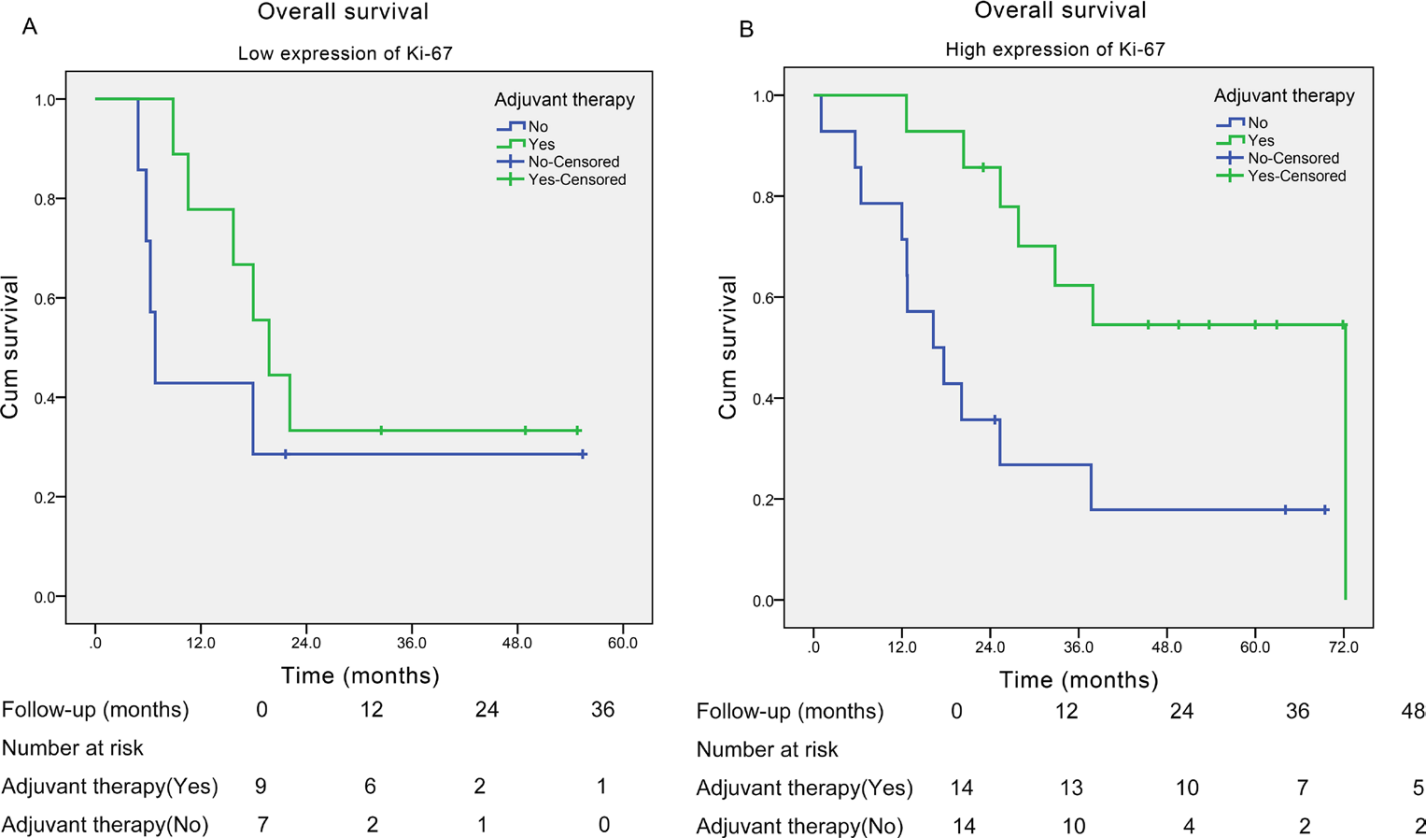
Kaplan-Meier curves of the overall survival of esophageal small cell carcinoma patients with different Ki-67 index stratified by adjuvant therapy (**A**) In patients with low expression of Ki-67 (≤ 50%) (*P* = 0.289); (**B**) in patients with high expression of Ki-67 (> 50%) (*P* = 0.008).

Since there was no obvious correlation between Ki-67 expression and clinicopathological characteristics of SCCE (including TNM stage), we therefore investigated further on the role of adjuvant therapy in both high Ki-67 index group and low Ki-67 index group. We found that in patients with low Ki-67 index (≤ 50%), patients receiving adjuvant therapy did not yield significantly longer survival time compared with those without adjuvant therapy (median survival time: 19.8 and 6.8 months, respectively; *P* = 0.289; Figure 3A ), while in patients with high Ki-67 index (> 50%), patients receiving adjuvant therapy obtained significantly longer survival time than those without adjuvant therapy (median survival time: 72.2 and 16.3 months, respectively; *P* = 0.008; Figure 3B).

## DISCUSSION

SCCE was an aggressively malignant tumor which originated from esophagus. In 2010 WHO classification [2] for digestive system neuroendocrine neoplasms, SCCE was defined as high grade (G3) neuroendocrine carcinoma with a mitotic count of > 20 per 10 high power fields and/ or a Ki-67 index > 20%. Previous studies have reported a median survival time of about 8.0 to 28.5 months for SCCE patients [13], indicating a poor prognosis. Due to its rarity, the prognostic factors as well as optimal therapy of SCCE were still hardly established. In our previous study, we have demonstrated that surgery with adjuvant therapy could be a preferred option for limited stage SCCE, especially for patients with a I/II TNM stage [13]. However, the prognostic factors of SCCE were rarely investigated, especially the prognostic value of immunohistochemical characteristics of SCCE.

Ki-67 is a nuclear protein expressed in the phases of cell cycle but not in the quiescent state (G0) [14]. Therefore, the expression of Ki-67 could be applied to assess tumor proliferation in clinical pathology, and Ki-67 labeling index (the fraction of Ki-67-positive tumor cells by immunohistochemistry) was widely explored for its prognostic value in various tumors including neuroendocrine neoplasms [15]. Moreover, Ki-67 index was also applied to neuroendocrine neoplasms for classification and grading in the 2010 WHO classification system [2]. Even though it is known that SCCE was characterized as a Ki-67 index of more than 20%, it is not known whether higher expression of Ki-67 (more than 50%) could serve as a prognostic factor for SCCE patients. Therefore, in this study, we tried to explore the prognostic role of Ki-67 in SCCE, and to our best knowledge, this is the first study to explore the prognostic value of immunohistochemical characteristic of SCCE.

We retrospectively reviewed and collected the clinicopathological and immunohistochemical data of patients who underwent esophagectomy and lymphadenectomy for SCCE in our department from January 2009 to January 2015. A total of 44 SCCE patients (accounting for 1.4% of all esophageal cancers) were finally analyzed in our current study. The baseline data of those SCCE patients shared similar characteristics with previously published studies [16–18], and we have discussed thoroughly in our previous study (see [13] for detailed discussion). Here in our current study, we only focus on the discussion of the immunohistochemical data of Ki-67 and its prognostic value for SCCE. After a median follow-up time of 54.8 months, the median survival time for the whole patients was 22.1 months, which indicated a poor prognosis of SCCE. In the prognostic analysis, only TNM stage, adjuvant therapy and Ki-67 index were found to be the independent prognostic factors for SCCE. Early TNM stage, adjuvant therapy, and high expression of Ki-67 (> 50%) were found to be prognosticators of favorable survival of SCCE patients with surgical resection. However, Ki-67 index was found to have no obvious correlation with any clinicopathological characteristics, including pT stage and pN stage. Our subgroup analysis found that in high Ki-67 expression group adjuvant therapy yielded significant survival benefit, while in low Ki-67 expression group adjuvant did not bring significant survival benefit. Therefore, it seemed that high Ki-67 index (> 50%) could not only serve as an independent favorable prognostic factor but also an indicator of survival benefit from adjuvant therapy for SCCE.

Because of the role of proliferation marker, Ki-67 has been widely investigated as prognostic marker for various tumors [4, 15, 19]. In most cases, high expression of Ki-67 was found to be associated with poor survival of tumor patients [7–10]. For example, in esophageal squamous cell carcinoma, high expression of Ki-67 correlated with significantly shorter overall survival [20]. Even in neuroendocrine tumors, Ki-67 expression was found to have negative impact on prognosis, with higher level of expression representing increasingly poor survival [7]. Moreover, Sorbye et al. [21] also showed that gastrointestinal neuroendocrine carcinoma patients with a Ki-67 index of less than 55% had longer survival than patients with a Ki-67 index of more than 55% (*P* < 0.05). It is reasonable that higher Ki-67 expression correlated with worse prognosis, because previous studies have shown that high proliferative index (high Ki-67 expression) may be related to aggressive behavior [22] and that Ki-67 antigen may play a role in promoting cell proliferation through various mechanisms [23]. However, contrast to above studies, in our study, we found that in SCCE patients, high expression of Ki-67 (> 50%) was significantly related to longer survival compared with low expression of Ki-67 (≤ 50%). Similar results could also be observed in colorectal cancer [6] and even in esophageal adenocarcinoma [24], but the reason for this discrepancy between Ki-67 expression on prognosis of patients with different cancers remains unclear. However, our further analysis for the role of adjuvant therapy in patients with different Ki-67 expression level found that patients with high Ki-67 index obtained significant survival benefit from adjuvant therapy, indicating that SCCE with high Ki-67 expression responded better to adjuvant therapy (chemoradiotherapy). Previously, Kitamura et al. [25] found that esophageal cancer patients with high Ki-67 index obtained significantly higher efficacy rate than patients with low Ki-67 index (73.9% versus 38.5%, *P* = 0.0013) after receiving neoadjuvant chemoradiotherapy. Lam et al. [22] also suggested high proliferative index in esophageal small cell carcinoma may be related to high sensitivity to chemotherapy and radiotherapy. Moreover, Sorbye et al. [21] found that high expression of Ki-67 was significantly related with high response rate of chemotherapy in gastrointestinal neuroendocrine carcinoma (42% versus 15%, *P* < 0.001). Therefore, the evidence that high expression of Ki-67 was related to high sensitivity to chemoradiotherapy [21, 22] may to some degrees explain the favorable prognosis of high expression of Ki-67 in SCCE after surgical resection, as more than half of those patients received adjuvant therapy. However, more studies are needed to explored the detailed mechanisms of the predictive role of Ki-67 in SCCE as well as other cancer types. In conclusion, our study provided the initial evidence that Ki-67 could serve as a prognostic factor for SCCE, and more importantly, Ki-67 expression level could also serve as an indication of providing adjuvant therapy for SCCE patients with surgical resection in clinical practice.

### Limitations

Even though we investigated the prognostic value of Ki-67 in SCCE for the first time, there were several limitations in our study. First, our retrospective study design has its innate limitation of analytical power, and some important data such as the detailed information of adjuvant therapy and other molecular and genetic markers were omitted due to lack of sufficient data. Second, we drew above conclusions based on a small sample size of 44 cases, especially in the subgroup analysis of correlation between adjuvant therapy and Ki-67 index, which may limit our validity of overall results. Moreover, in this study, we only focused on the immunohistochemical profile of Ki-67 in SCCE, while prognostic values of other molecular and genetic markers were also worthy of further investigation for future relevant studies. Finally, because of relatively early preoperative stage, none of these patients received neoadjuvant therapy, and therefore the predictive role of Ki-67 in SCCE treated with neoadjuvant therapy and surgery remains unclear. Therefore, even though our study provided primary evidence for the prognostic role of Ki-67 for SCCE, our results should be interpreted with cautions due to above limitations. Thus further relevant studies are badly needed to update and confirm our current conclusions.

## MATERIALS AND METHODS

### Patients

We retrospectively collected the data of patients with pathologically and immunohistochemically diagnosed SCCE who underwent esophagectomy with two-field lymphadenectomy in our department from January 2009 to January 2015 from our database. This study was approved by the Ethics Committee of West China Hospital, Sichuan University (No.20161011). Because our study was a retrospective prognosis analysis and analyzed anonymously, the ethics committee waived the need for informed consents from those patients.

### Data for analysis

Demographic data, pathologic findings, tumor stage, adjuvant therapy, and survival time as well as Ki-67 index were collected for analysis. The pathologic findings consisted of homology of the SCCE, lymphovascular invasion, and tumor location. The 2009 AJCC TNM staging system for esophageal squamous cell carcinoma was applied for the pT and pN stage of those patient as described in our previous study [13]. Adjuvant therapy included chemotherapy, radiotherapy, and chemoradiotherapy. Our last follow-up was conducted by telephone, outpatient department visit, or even patient visiting in October 2016. Survival time was calculated from the day of operation to the date of death or last follow-up. Ki-67 index was conducted by immunohistochemistry of Ki-67 antigen stained by Ki-67 antibody demonstrated as follow by the pathologist from our hospital.

### Ki-67 immunohistochemistry

The immunohistochemistry stain methods of Ki-67 were similar to previous study [26]. The resected tumor tissues were sectioned at a thickness of 4 μm after formalin fixing and paraffin embedding. And then, the Ventana automated immunostainer was used to stain the sections with an antibody for Ki-67 (Dako, Carpintera CA, monoclonal MIB-1, dilution 1:100), and the OptiView DAB IHC detection kit and the OptiView Ampi cation kits were used according to the manufacturer's instructions. Positive immunostaining was only nuclear staining in heterocytes (Epithelial cells of basal layer could be positive control).

**Figure 4:**
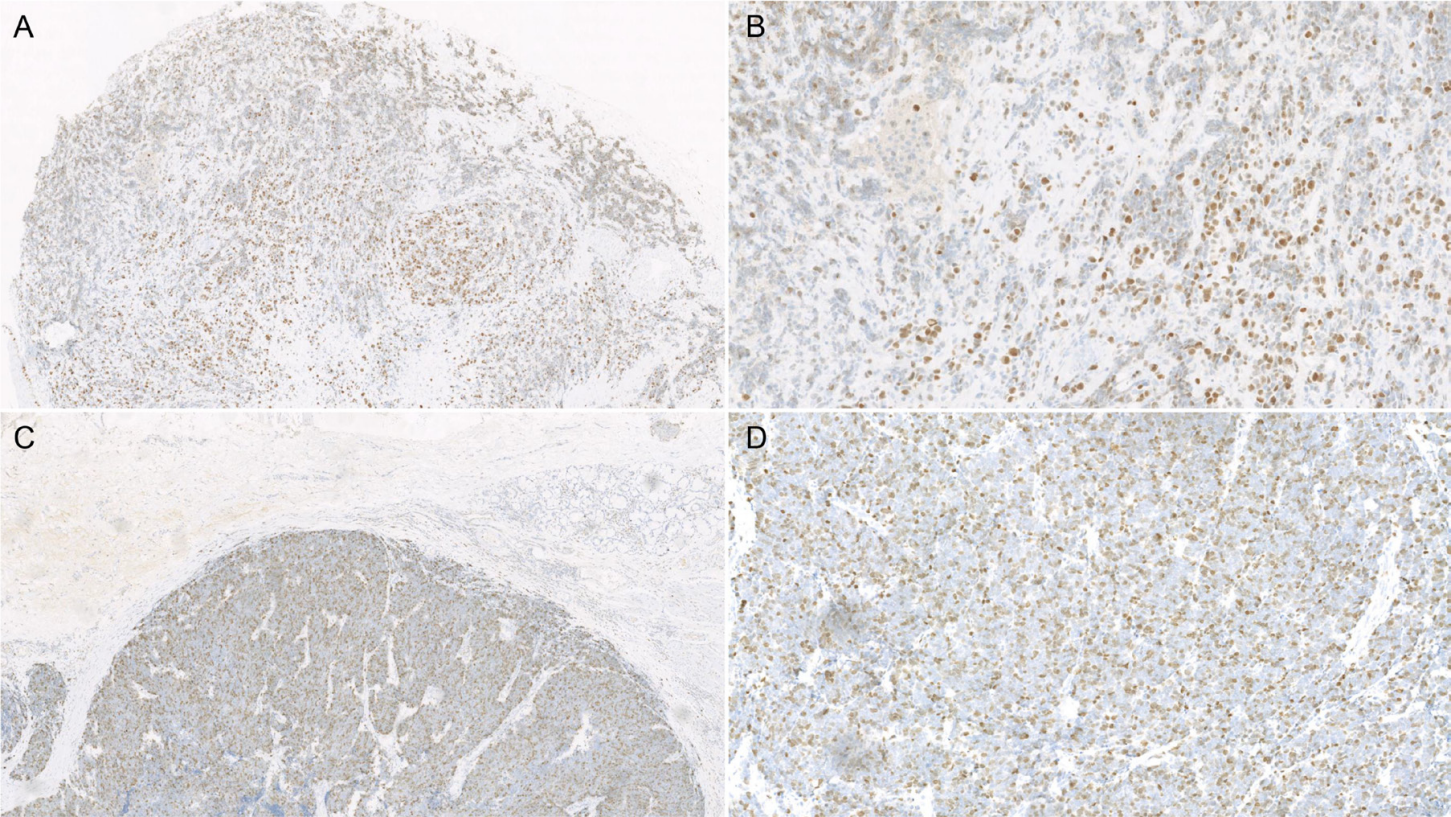
Microscope photographs of immunohistochemistry stain of Ki-67 expression in esophageal small cell carcinoma: Low expression of Ki-67 (< 50%, A: ×40; B: ×200) and high expression of Ki-67 (> 50%, C: ×40; D: ×200)

For statistical analysis, the immunostaining results of Ki-67 were categorized into two groups according to the percentage of Ki-67-positive tumor cells per high power fields: low Ki-67 index group (≤ 50%) and high Ki-67 index group (more than 50%). (Figure 4).

### Statistics

Statistical analysis was performed by using SPSS 19.0 software (SPSS Corp, Chicago, IL, USA). Data were represented as the mean ± standard deviation for continuous variables or number (%) for categorical data. Independent sample *t*-test or the Mann-Whitney test was applied for comparison of continuous data between groups, and chi-square test or Fisher's exact test was applied for comparison of categorical data. The survival time was calculated by the Kaplan-Meier analysis, and the log-rank test was used to estimate the association between eligible variables and survival time. The Cox's hazard regression model was applied to explore independent prognostic factors for SCCE patients. A two-sided probability value of less than 0.05 was considered statistically significant.

## CONCLUSIONS

In this study, we explored the prognostic value of Ki-67 expression in SCCE patients with surgical resection for the first time. We found that high expression of Ki-67 was significantly correlated to better prognosis of SCCE patients, especially for those with adjuvant therapy. Therefore, high expression of Ki-67 could not only serve as a favorable prognostic factor of SCCE patients with surgical resection but also an indication of providing adjuvant therapy for those with high expression of Ki-67.
